# *Candida auris* in Germany and Previous Exposure to Foreign Healthcare

**DOI:** 10.3201/eid2509.190262

**Published:** 2019-09

**Authors:** Axel Hamprecht, Amelia E. Barber, Sibylle C. Mellinghoff, Philipp Thelen, Grit Walther, Yanying Yu, Priya Neurgaonkar, Thomas Dandekar, Oliver A. Cornely, Ronny Martin, Oliver Kurzai, on behalf of the German Candida auris Study Group

**Affiliations:** German Centre for Infection Research, Cologne, Germany (A. Hamprecht, S.C. Mellinghoff, O.A. Cornely);; University of Cologne, Cologne (A. Hamprecht, O.A. Cornely);; Leibniz Institute for Natural Product Research and Infection Biology–Hans-Knoell-Institute, Jena, Germany (A.E. Barber, G. Walther, O. Kurzai);; University Hospital Cologne, Cologne (S.C. Mellinghoff, P. Thelen);; University of Würzburg, Würzburg, Germany (Y. Yu, P. Neurgaonkar, T. Dandekar, R. Martin, O. Kurzai)

**Keywords:** mycology, infectious diseases, *Candida auris*, Germany, fungi

## Abstract

The emerging yeast *Candida auris* has disseminated worldwide. We report on 7 cases identified in Germany during 2015–2017. In 6 of these cases, *C. auris* was isolated from patients previously hospitalized abroad. Whole-genome sequencing and epidemiologic analyses revealed that all patients in Germany were infected with different strains.

*Candida auris* is an emerging yeast that was initially described in 2009 after a case of otitis externa in Japan (*1*). Since then, healthcare-associated infections have been reported worldwide (*2*). *C. auris* has caused outbreaks in hospitals in Asia, Africa, and Latin America (*2*–*4*). In Europe, 620 *C. auris* cases were observed during 2013–2017 (24% infections, 76% colonizations), including 7 cases in Germany (*5*). Most *C. auris* isolates exhibit resistance to fluconazole, and susceptibility to other azoles, amphotericin B, and echinocandins varies among isolates. Some strains show resistance to all 3 classes of antifungal drugs (*6*). 

We report on the occurrence of *C. auris* in Germany and its link to prior healthcare exposure in the Middle East, Asia, Africa, or the United States. *C. auris* was isolated from 7 patients (4 male, 3 female, all in different, unrelated hospitals) during November 2015–December 2017 (Appendix Table). Six of the patients had previously been treated in healthcare centers outside Germany and were transferred to Germany for further treatment. No further suspicious cases or isolates were reported to the National Reference Centre for Fungal Infections (Jena, Germany); however, reporting is not mandatory, and the possibility of missed cases cannot be excluded. 

Of the 7 patients, 3 had been in isolation before detection of *C. auris* as a result of known colonization with carbapenemase-producing *Enterobacteriaceae*. No secondary *C. auris* cases were detected in any of the hospitals until March 2019. However, because no contact screening was performed, transmission resulting in asymptomatic carriage cannot be excluded.

Isolates from 6 patients were available for further testing. Biochemical identification of isolates by API ID 32C resulted in misidentification as *C. sake* (5 of 6) or *C. intermedia* (1 of 6). In contrast to previous versions, Vitek 2 version 08.01 (bioMérieux, https://www.biomerieux-diagnostics.com) identified all isolates as *C. auris* with 93%–99% likelihood. With VitekMS (bioMérieux) matrix-assisted laser desorption ionization time-of-flight (MALDI-TOF) mass spectrometry, no identification was achieved. However, a recent database update for VitekMS (version 3.2), which was not available at the time of our testing, corrected the identification failure in the VitekMS (data not shown). The Bruker Biotyper system (https://www.bruker.com) correctly identified all strains, albeit some with a low score (1.6–1.99). Whereas Bruker recommends that a score of 2.0 be used for species identification, a score >1.7 has been shown to be sufficient for reliable species identification (*7*). At the time of testing, Bruker’s research-use-only library did not include a *C. auris* strain of the South Asian clade, which most of the German isolates belong to. Because *C. auris* exhibits considerable heterogeneity of mass spectra between geographic clusters, this missing clade likely explains the low scores (*8*).

Molecular identification using internal transcribed spacer technology identified all *C. auris* strains with 100% identity to the reference strain DSM 21092/CBS 10913. For available isolates, we performed whole-genome sequencing and aligned reads to the B8441 v2 reference genome (Figure; Appendix). A phylogenetic tree generated from whole-genome single-nucleotide polymorphism (SNP) data indicated that the isolates NRZ-2015-214, NRZ-2017-288, NRZ-2017-367, NRZ-2017-394/1-2, and NRZ-2017-505 belong to the South Asian clade, whereas NRZ-2018-545 was related to the African clade (Figure). In line with previous studies, the genetic differences observed between isolates of the same clade were small (30–800 SNPs), whereas differences between clades were large (36,000–147,000 SNPs) (*4*,*9*). Whole-genome data show that all cases identified in Germany harbor unique isolates, thus excluding transmission between these patients (Figure). As a control, the clonality of serial isolates NRZ-2017-394/1 and NRZ-2017-394/2, taken from the same patient on 2 different occasions, was confirmed; the 2 isolates were separated by only a single SNP (Figure).

**Figure:**
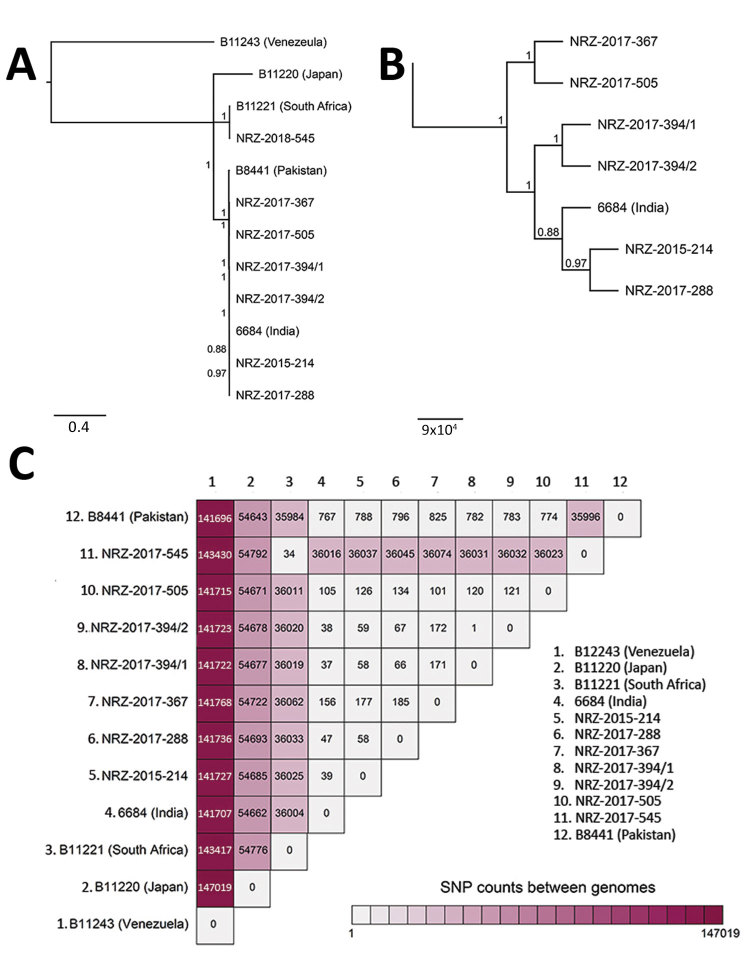
Genetic relationships of *Candida auris* isolates based on whole-genome sequencing SNP analysis. A) Maximum-likelihood phylogeny of *C. auris* isolates from Germany (indicated by NRZ prefix) inferred to reveal a possible geographic origin. The isolates were contrasted against strains representing the 4 different clades of *C. auris*: South American (strain B114243 from Venezuela), East Asian (B11220 from Japan), South African (B11221 from South Africa), and South Asian (B8441 from Pakistan and 6684 from India). B) Higher resolution of the tree shown in panel A to better visualize the relationship between the isolates belonging to the South Asian clade. Scale bars in panels A and B indicate nucleotide substitutions per site. C) SNP counts between the genomes of the isolates from Germany and the representative strains from the different clades. SNP, single-nucleotide polymorphism.

MICs of fluconazole were high for all isolates (>64 mg/L), wheareas the MICs for other antifungals were variable (Appendix Table). With the exception of NRZ-2017-545, all isolates carried either the Y132F or the K143R mutations in the *ERG11* gene. However, although these mutations are linked to azole resistance, they did not result in elevated MICs for all azoles in these isolates (Appendix Table) (*10*). We identified a S639Y mutation in the *FKS1* hotspot 1 of isolate NRZ-2017-505, which was highly resistant to anidulafungin (MIC 16 mg/L).

In conclusion, *C. auris* has so far been isolated from individual cases in Germany. Most of these cases (6 of 7) occurred in patients who had previously been hospitalized abroad and were admitted to hospitals in Germany for continuation of medical treatment. No information on contact or environmental screening was available; such screening was likely not performed in most institutions. Thus, silent transmission and resulting carriage may have occurred unnoticed. However, no secondary cases were detected in any of the 7 hospitals affected.

Appendix Discussion of materials and methods and clinical case presentations for the study of *Candida auris* and previous healthcare in Germany.
