# Supplementary material for: Candida auris in Germany and Previous Exposure to Foreign Healthcare
Source: Emerg Infect Dis. 2019 Sep;25(9):1763–5. doi: 10.3201/eid2509.190262 (PMC6711216; doi:10.3201/eid2509.190262)
Supplement: Appendix — Discussion of materials and methods and clinical case presentations for the study of Candida auris and previous healthcare in Germany. [file 19-0262-Techapp-s1.pdf]

Article DOI: https://doi.org/10.3201/eid2509.190262

Candida auris in Germany and Previous Exposure to Foreign Healthcare

Appendix

Materials and Methods

Isolates

Since the worldwide emergence of *Candida auris*, the National Reference Centre for Fungal Infections (NRZMyk) has issued several alerts on this species and informed diagnostic laboratories in Germany to send all suspicious *Candida* spp. isolates to the reference laboratory for identification. In total, during November 2015–December 2017, we collected 8 isolates from 6 patients; for 1 case (reliable identification with Bruker MALDI), no isolate was available.

Identification and Susceptibility Testing

We identified all isolates by biochemical methods (Vitek2 Yeast Card, API ID32C V4.0 (bioMérieux) and by MALDI-TOF using 2 different systems (VitekMS, bioMérieux or Biotyper Microflex, Bruker Daltonics) and 2 protein extraction protocols, as previously described (*1*). In addition, we identified all isolates by PCR and sequencing of the internal transcribed spacer (ITS) (*2*).

The isolates were tested by broth microdilution according to European Committee on Antimicrobial Susceptibility Testing (EUCAST) standards and minimal inhibitory concentrations (MIC) for fluconazole, itraconazole, posaconazole, voriconazole, anidulafungin, and amphotericin B were determined (EUCAST E.DEF 7.3.1. 2017, www.eucast.org).

DNA Isolation

We isolated fungal DNA with a phenol-free procedure, as described previously (*3*). We analyzed the quality of the isolated DNA by Nanodrop and with an Invitrogen Qubit Fluorometer (https://www.thermofisher.com).

Whole-Genome Sequencing

Library preparation and whole-genome sequencing of *C. auris* isolates was performed by either GATC (Konstanz, Germany) or LGC Genomics (Berlin, Germany) on the Illumina platform using 2x 150-bp paired end reads. Illumina adapters were removed from the GATC-sequenced isolates using Trim Galore version 0.4.3.1 (Babraham Institute, https://www.babraham.ac.uk). Further data processing was performed using the CSI Phylogeny Pipeline (*4*). Briefly, reads were mapped to the B8441 v2 reference genome using BWA version 0.7.12 (*5*). SNP calling was performed using SAMtools version 0.1.18 (*6*). Following variant detection, SNPs were filtered for a minimum depth of 10, minimum relative depth of 10, minimum SNP quality of 30, and a Z-score of greater than 1.96. No SNP pruning (removal of closely spaced SNPs) was performed to better separate closely related isolates. SNPs were then concatenated and maximum-likelihood phylogeny inferred using FastTree version 2.1.7 (*7*). For comparison of the isolates in this study to reference strains from other *C. auris* clades, raw sequence data from the Indian isolate 6684 (BioSample ID SAMN03200169), the Japanese isolate B11220 (BioSample ID SAMN05379608), the South African isolate B11221 (BioSample ID SAMN05379609), and the Venezuelan isolate B11243 (BioSample ID SAMN05379619) were downloaded from the National Center for Biotechnology Information (NCBI) and processed using the same pipeline.

Data Availability

Raw sequence read files were uploaded to the NCBI Sequence Read Archive and are publicly available under BioProject IDs PRJNA485145, PRJNA485239, PRJNA485259, PRJNA485409, PRJNA485414, and PRJNA485415.

Clinical Case Presentations

Patient 1 was admitted for a prosthetic joint infection, which was first diagnosed and treated with antimicrobial drugs in Oman. After hospital admission in Germany and replacement of the prosthesis, a febrile episode occurred and *C. auris* was isolated from blood cultures. The patient recovered after treatment with an echinocandin and *C. auris* has not been isolated in subsequent hospital stays.

Patient 2 was treated in the intensive care unit for systemic inflammatory response syndrome. *C. auris* was detected incidentally when the tip of the central venous catheter was sent to the microbiology department as part of the clinical routine. The patient did not receive any antifungal treatment, as no clinical manifestation of *C. auris* infection was observed; the patient is still alive. This patient had no known history of travel or hospitalization abroad within the past year.

Patient 3 had an intracranial hemorrhage that had previously been treated in a Saudi Arabian healthcare facility. Because of a known colonization with carbapenemase-producing Enterobacteriaceae, the patient was placed in a single room. Culture of the catheter urine revealed growth of 10^5 CFU/mL of *C. auris*. Even though urinary catheters were changed several times, *C. auris* was isolated from 2 follow-up urine specimens. Furthermore, *C. auris* was grown from the groin and the tracheostomy. In the absence of any signs of infection in the later course, the patient was transferred to rehabilitation without further treatment of *C. auris*.

Patient 4 was transferred to Germany from Dubai because of a neurologic disorder. *C. auris* was isolated from catheter urine. No treatment was initiated, as the patient was asymptomatic and was discharged alive.

Patient 5 had a multiple trauma, which was initially treated in Russia. From an intraabdominal sample taken during surgery, *Enterococcus faecalis, E. avium,* and *C. auris* were isolated. Treatment with ampicillin–sulbactam and amphotericin B was initiated. The patient was transferred to another hospital in good condition 10 days after surgery.

Patient 6 was treated in Germany for sequelae of an injury originating in Afghanistan. Because of systemic signs of infection, blood cultures were drawn, revealing the presence of *C. auris*. Infection control measures were implemented for this patient after detection of *C. auris*. Because the isolate had elevated MICs for echinocandins, treatment with liposomal amphotericin B and voriconazole was initiated. *C. auris* was also isolated from the urine, likely as a result of an infection of the renal pelvis and the presence of several large kidney stones. Candiduria has not improved for >6 months despite prolonged therapy with voriconazole and amphotericin B. However, since >6 months, no *C. auris* is detectable from this patient’s specimen.

Patient 7 was admitted for the treatment of tetraplegia of unknown origin. In the personal history of the patient, previous hospitalizations were recorded in Kenya (2017), Germany (2016),

the United States (2015), and the UK (2014). *C. auris* was grown from the urine but was not treated, as there were no symptoms of a urinary tract infection. The patient was still alive as of November 2018. No further information is available.

References

1. Hamprecht A, Christ S, Oestreicher T, Plum G, Kempf VA, Göttig S. Performance of two MALDI-TOF MS systems for the identification of yeasts isolated from bloodstream infections and cerebrospinal fluids using a time-saving direct transfer protocol. Med Microbiol Immunol (Berl). 2014;203:93–9. http://dx.doi.org/10.1007/s00430-013-0319-9

2. White TBT, Lee S, Taylor JW. Amplification and direct sequencing of fungal ribosomal RNA genes for phylogenetics. In: Innis MA, Gelfand DH, Sninsky JJ, White TJ, editors. PCR protocols: a guide to methods and applications. New York: Academic Press; 1990. p. 315–22.

3. Walther G, Pawłowska J, Alastruey-Izquierdo A, Wrzosek M, Rodriguez-Tudela JL, Dolatabadi S, et al. DNA barcoding in Mucorales: an inventory of biodiversity. Persoonia. 2013;30:11–47. http://dx.doi.org/10.3767/003158513X665070

4. Kaas RS, Leekitcharoenphon P, Aarestrup FM, Lund O. Solving the problem of comparing whole bacterial genomes across different sequencing platforms. PLoS One. 2014;9:e104984. http://dx.doi.org/10.1371/journal.pone.0104984

5. Li H, Durbin R. Fast and accurate short read alignment with Burrows-Wheeler transform. Bioinformatics. 2009;25:1754–60. http://dx.doi.org/10.1093/bioinformatics/btp324

6. Li H, Handsaker B, Wysoker A, Fennell T, Ruan J, Homer N, et al.; 1000 Genome Project Data Processing Subgroup. The Sequence Alignment/Map format and SAMtools. Bioinformatics. 2009;25:2078–9. http://dx.doi.org/10.1093/bioinformatics/btp352

7. Price MN, Dehal PS, Arkin AP. FastTree 2—approximately maximum-likelihood trees for large alignments. PLoS One. 2010;5:e9490. http://dx.doi.org/10.1371/journal.pone.0009490

Appendix Table. Characteristics of patients and *Candida auris* isolates, Germany, 2015–2017*

	C. auris isolates			Patient details			Antifungal susceptibility, μg/mL†						Resistance mutations‡		
Patient no.	Date of isolation	Isolate no.	Genetic clade	Isolation site	Age, y/sex	Previous hospitalization	FLU	ISA	ITR	POS	VOR	ANI	*ERG11* mutations	*FKS1* HS1	*FKS1* HS2
1	2015 Nov	NRZ 2015-214	South Asian	Blood culture	65/M	Oman	≥64	ND	0.05	0.03	1	0.25	Y132F	None	None
2	2015 Dec	NA§	NA§	CVC tip	50/F	None	≥64	0.03	0.05	0.06	1	0.125	Y132F	None	None
3	2017 Jun	NRZ 2017-394	South Asian	Catheter urine	67/F	Saudi Arabia	≥64	1	2	0.5	2	0.25	K143R	none	None
4	2017 Jul	NRZ 2017-288	South Asian	Catheter urine	48/F	Dubai	≥64	≤0.016	0.25	≤0.016	0.25	0.125	Y132F	None	None
5	2017 Aug	NRZ 2017-367	South Asian	Intraoperative tissue sample, pelvis	57/M	Russia	≥64	≤0.016	0.25	≤0.016	0.5	0.125	Y132F	None	None
6	2017 Nov	NRZ 2017-505	South Asian	Blood culture	15/M	Afghanistan	≥64	0.25	0.5	≤0.016	0.5	16	K143R	S639Y	None
7	2017 Dec	NRZ 2017-545	African	Urine	60/M	Great Britain, USA, Kenya	≥64	≤0.016	0.25	0.06	2	0.5	V124A F125L	None	None

*ANI, anidulafungin; CVC, central venous catheter; FLU, fluconazole; ISA, isavuconazole; ITR, itraconazole; NA, not available; ND, not determined; POS posaconazole; VOR, voriconazole.
† Results of microdilution tests according to European Committee on Antimicrobial Susceptibility Testing (EUCAST) recommendations (*www.eucast.org).*
‡ERG11 was completely sequenced, whereas for FKS1 only hotspot 1 and 2 were sequenced. If existing, only mutations in the protein sequence are shown.
§The isolate was reliably identified as *C. auris* using Bruker mass spectroscopy but was not stored for further analysis; thus, no information on clade is available. Susceptibility testing was performed using the Micronaut system (Merlin Diagnostika, https://www.merlin-diagnostika.de) but could not be confirmed using a reference methodology.
